# Supplementary material for: Insights into the Cytochrome P450 Monooxygenase Superfamily in Osmanthus fragrans and the Role of OfCYP142 in Linalool Synthesis
Source: Int J Mol Sci. 2022 Oct 12;23(20):12150. doi: 10.3390/ijms232012150 (PMC9602793; doi:10.3390/ijms232012150)
Supplement: Supplementary file 1 [file ijms-23-12150-s001.zip › Supporting information for publication .pdf]

SUPPLEMENTARY MATERIALS

Insights into the Cytochrome P450 Monooxygenase Superfamily in *Osmanthus fragrans* and the Role of *OfCYP142* in Linalool Synthesis

Jiawei Liu†‡, Hongmin Hu†‡, Huimin Shen†‡, Qingyin Tian†‡, Wenjie Ding†‡, Xiulian Yang†‡, Lianggui Wang†‡, Yuanzheng Yue†‡*

†Key Laboratory of Landscape Architecture, Jiangsu Province, College of Landscape Architecture, Nanjing Forestry University, Nanjing 210037 Nanjing, China

‡ Co-Innovation Center for Sustainable Forestry in Southern China, Nanjing Forestry University, Nanjing 210037 Nanjing, China

* Correspondence: yueyuanzheng@njfu.edu.cn; Tel.: +86-27-85428770

Supplementary table legends

Table S1 List of the 276 *OfCYP* genes identified in this study.

Table S2 CYP proteins represented in phylogenetic analysis.

Table S3 Analysis and distribution of conserved motifs in Sweet Osmanthus P450 proteins.

Table S4 The FPKM values of *OfCYP* genes in different *O. fragrans* organs.

Table S5 The FPKM values of *OfCYP* genes exposed to cold treatment (4°C) for 120 h.

Table S6 The expression values of *OfCYP* genes in response to salt treatment for 72 h.

Table S7 The expression values of *OfCYP* genes in at five flowering stages of *O. fragrans*.

Table S8 Identity and quantity of volatile compounds with relative content in the transgenic petals.

Table S9 Information about 94 KEGG pathway which DEGs enriched in.

Table S10 Identity and quantity of volatile compounds with relative content in

the transgenic petals.

Table S11 Information about *P450* genes in plants.

Table S12 Primers used for RT-qPCR analysis, OfCYP142 cloning and its vector construction.

Supplementary figure legends

Figure S1 Gene structure and motif analysis of the *OfCYP* genes.

Figure S2 Expression profiles of OfCYPs in different tissues and at different stages of flower development.

Figure S3 Expression levels of *OfCYP*s in response to cold and salt stress conditions.

Figure S4 Gas chromatography traces of Agrobacterium-infiltrated *O. fragrans* petals transiently expressing *OfCYP142* and empty vector respectively.

Figure S5 Score scatter plot of the RNA-seq profiles of two types flowers with overexpression of *OfCYP142* and empty vector in *O. fragrans*.

Figure S6 KEGG pathway analysis of differentially expressed genes. The advanced bubble chart shows enrichment of differentially expressed genes in certain pathways.

Figure S7 Gas chromatography traces of Agrobacterium-infiltrated *N. tabacum* flowers stably expressing *OfCYP142* and empty vector respectively.

Figure S1. Gene structure and motif analysis of the *OfCYP* genes. (A) Phylogenetic tree of 276 OfCYP proteins. (B) Motif composition of OfCYP proteins. The MEME tool was used to predict motifs, and 20 motifs are shown in different colors. The detailed information of the 20 motifs is provided in Table S3. (C) Exon–intron structure of *OfCYP* genes. Green boxes and black lines represent exons and introns, respectively.

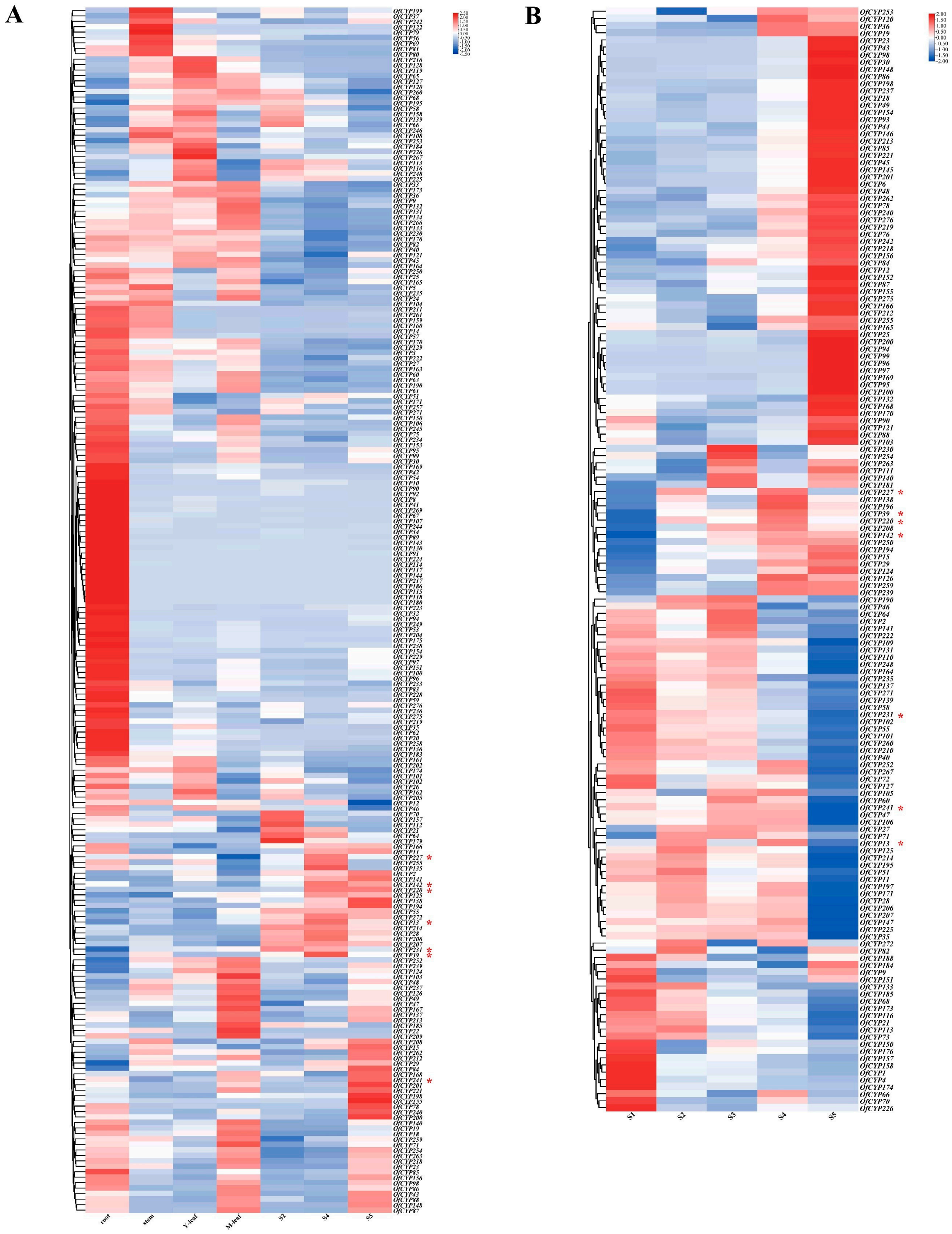

Figure S2. Expression profiles of OfCYPs in different tissues and at different stages of flower development. (A) Transcription levels of OfCYPs in different tissues. The different tissues were root, stem, and leaf (Y-leaf, young leaf; M-leaf, mature leaf); flower (S2, bud-eye stage; S4, full blooming stage; S5, flower fading stage). (B) Transcription levels of OfCYPs in five distinct stages of flower development: S1, bud-pedicel stage; S2, bud-eye stage; S3, primary

blooming stage; S4, full blooming stage; and S5, flower fading stage. The RPKM values obtained from the transcriptome data and FPKM (Fragments Per Kilobase of exon model per Million mapped fragments) values derived from the RNA-seq data in five distinct stages of flower development of OfCYPs were normalized by log2 transformation. The heatmap was generated by TBtools software.

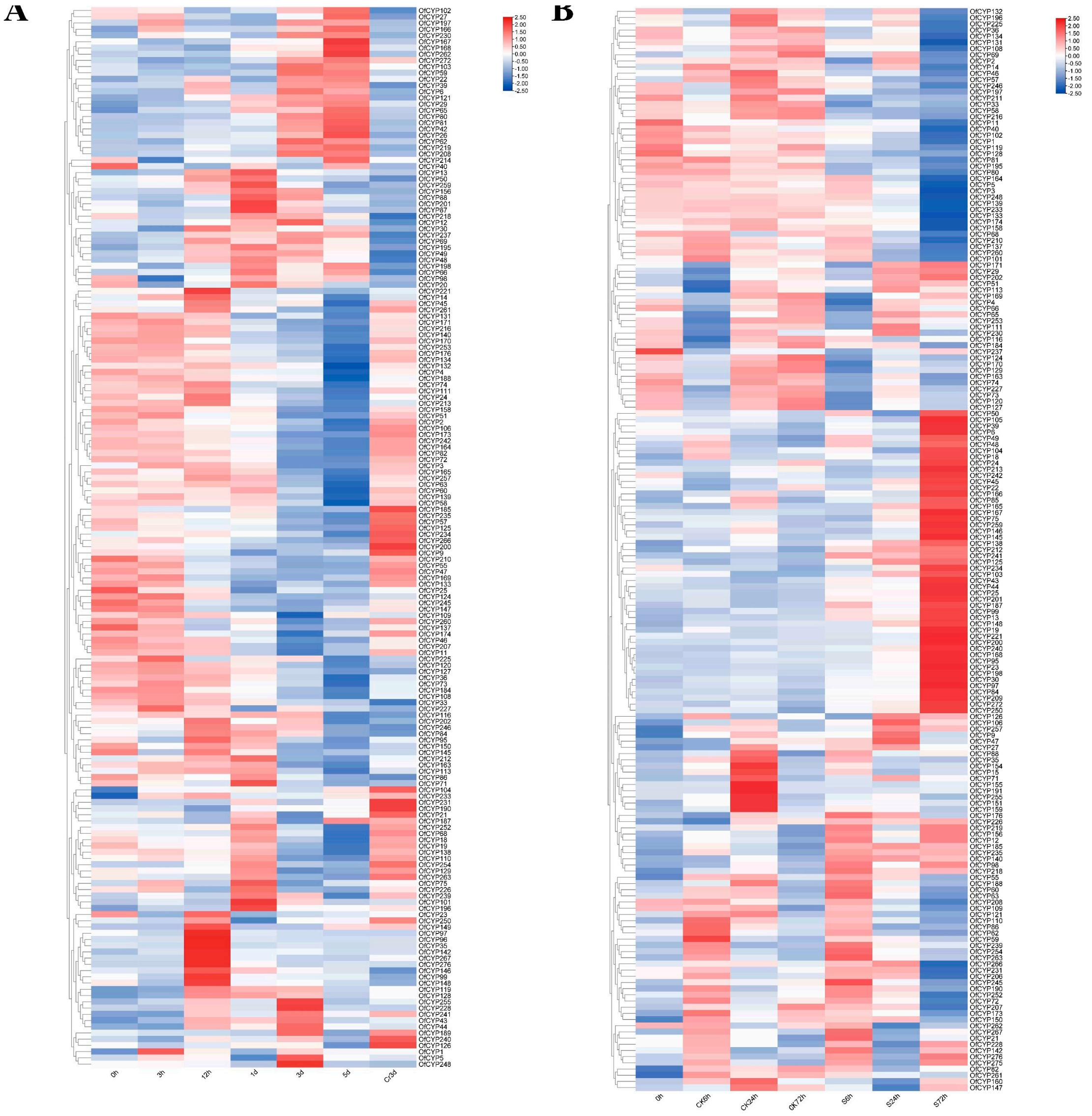

Figure S3. Expression levels of *OfCYP*s in response to cold and salt stress conditions. (A) Expression profiles of *OfCYP*s in response to cold stress. 0 h, 3 h, 12 h, 24 h, 72 h, and 120 h

represent the different times that the cold treatment was maintained. The Cr72 h represents recovery for 72 h after a 120-h cold treatment. (B) Expression profiles of *OfCYP*s in response to salt stress. S0, S6, S24, and S72 denote salt stress for up to 0 (control), 6, 24, and 72 h, respectively. The FPKM values for *OfCYP*s in leaves were obtained from the sweet osmanthus RNA-seq data under cold and salt stress conditions (Table S5 and Table S6) and were normalized by $\log_2$ transformation. The heatmap was generated by TBtools software.

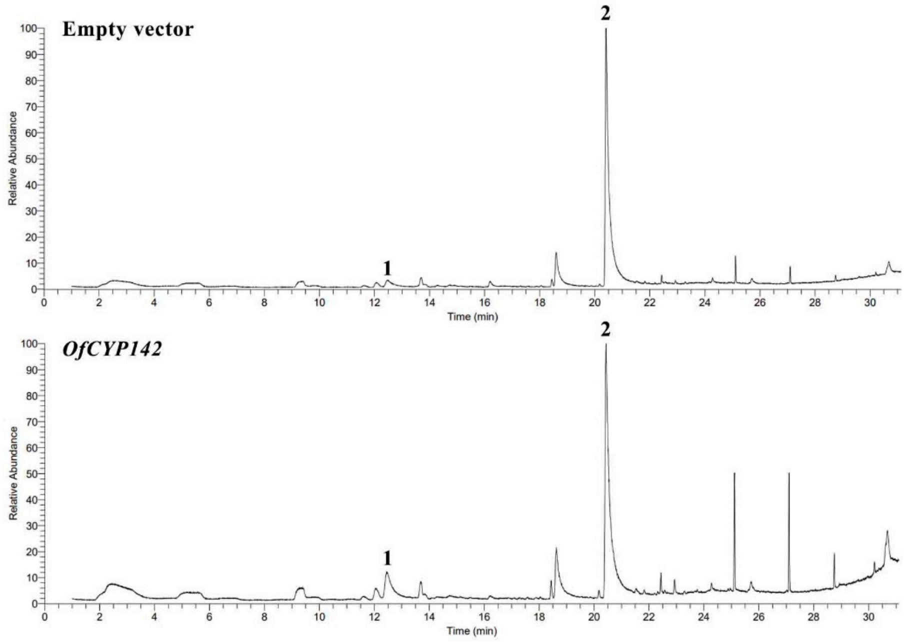

Figure S4. Gas chromatography traces of Agrobacterium-infiltrated *O. fragrans* petals transiently expressing *OfCYP142* and empty vector respectively. The identified compound peaks were 1. Linalool, 2. Ethyl caprate (Standard), respectively. The detailed information of the identified volatile compounds is provided in Table S8.

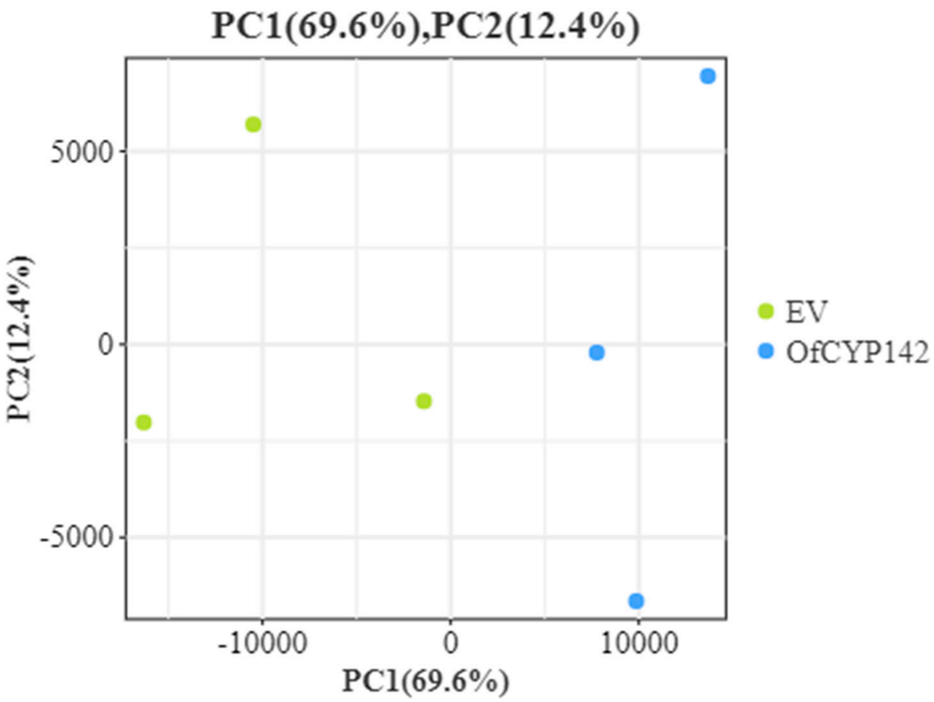

Figure S5. Score scatter plot of the RNA-seq profiles of two types flowers with overexpression of *OfCYP142* and empty vector in *O. fragrans*.

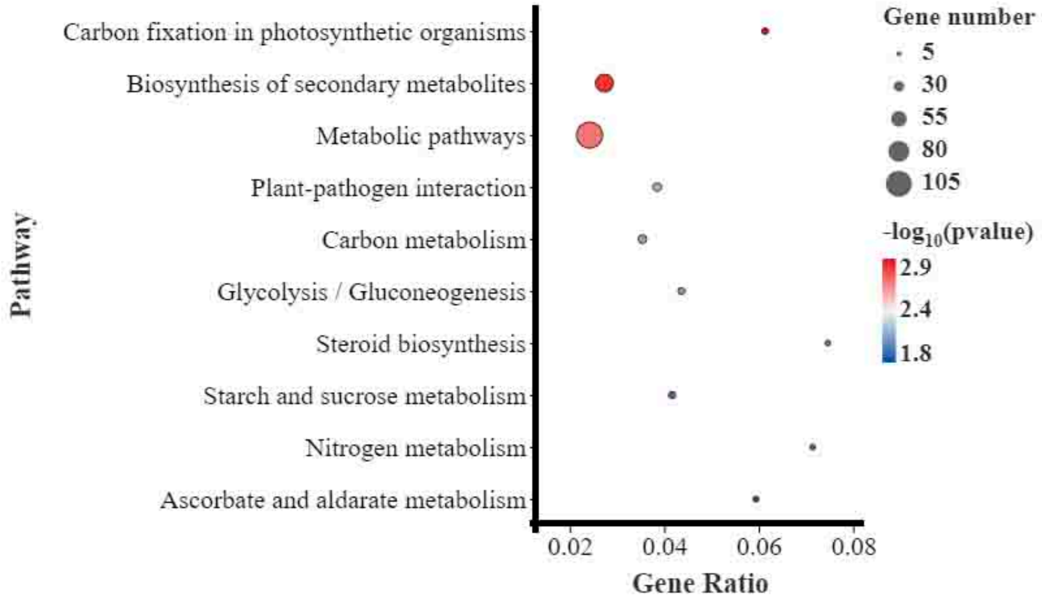

Figure S6. KEGG pathway analysis of differentially expressed genes. The advanced bubble chart shows enrichment of differentially expressed genes in certain pathways.

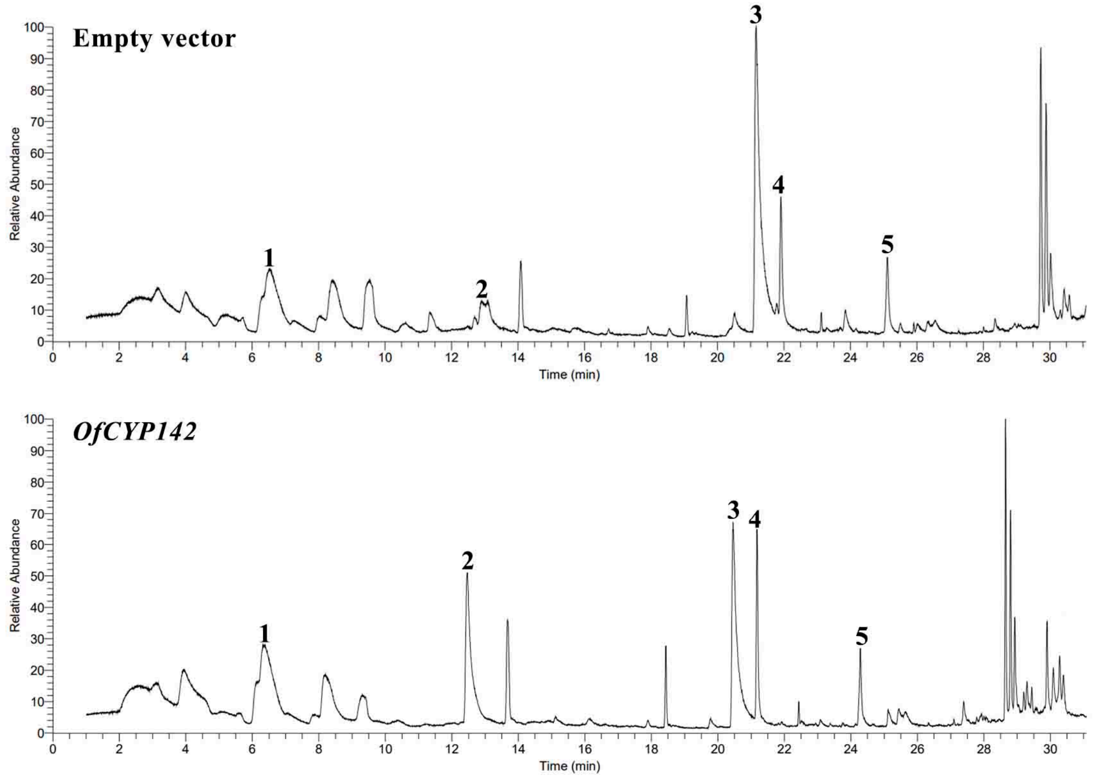

Figure S7. Gas chromatography traces of Agrobacterium-infiltrated *N. tabacum* flowers stably expressing *OfCYP142* and empty vector respectively. The identified compound peaks were 1. Cis-3-Hexen-1-ol, 2. Linalool, 3. Ethyl caprate (Standard), 4. (-)-β-caryophyllene, and 5. (-)-Caryophyllene oxide, respectively. The detailed information of the identified volatile compounds is provided in Table S10.
